# Supplementary material for: Janus effect of glucocorticoids on differentiation of muscle fibro/adipogenic progenitors
Source: Sci Rep. 2020 Mar 24;10:5363. doi: 10.1038/s41598-020-62194-6 (PMC7093513; doi:10.1038/s41598-020-62194-6)
Supplement: Supplementary file 1 — Supplementary material. [file 41598_2020_62194_MOESM1_ESM.docx]

**Supplementary Data**

**Janus effect of glucocorticoids on differentiation of muscle fibro/adipogenic progenitors**

Andrea Cerquone Perpetuini^1,*,#^, Giulio Giuliani^1,#^ Alessio Reggio^1^, Mauro Cerretani^3^, Marisabella Santoriello^3^, Roberta Stefanelli^1^, Alessandro Palma^1^, Simone Vumbaca^1^, Steven Harper^3,4^, Luisa Castagnoli^1^, Alberto Bresciani^3^ and Gianni Cesareni^1,2^.

^1^ Department of Biology, University of Rome “Tor Vergata”, Rome, Italy

^2^ Fondazione Santa Lucia Istituto di Ricovero e Cura a Carattere Scientifico (IRCCS), Rome, Italy

^3^ Department of Biology, IRBM S.p.A., via Pontina Km 30,600, 00071 Pomezia (Roma), Italy

^4^ Deceased

^*^ Corresponding author, email: andrea.cerquoneperpetuini@gmail.com

^#^ These authors contributed equally to this work

**Supplementary Figures Legends**

**Fig. S1. Flow cytometry analysis assessing FAPs purity.** (a) Scatter plots and histograms showing the expression of CD140a (PDGFRα) in SCA1-positive FAPs. The upper panels show unstained cells used as blank while in the lower panel cells were incubated in the presence of CD140-APC antibodies for 30 minutes. (b) Representative immunofluorescence analysis showing the expression of CD140a (PDGFRα, red) in SCA1- positive FAP preparations. Nuclei were stained with Hoechst 33342.

**Fig. S2. Anti-adipogenic compounds show an enrichment for GCs.** (a) Schematic representation of the screening results of the Prestwick chemical library. (b) Scatter plots representing cytotoxic, pro-adipogenic and anti-adipogenic compounds identified after the screening of the Prestwick library.

**Fig. S3. The expression of Gli genes is not induced upon treatment with budesonide.** (a) 3T3-L1 pre-adipocytes were expanded *in vitro* and incubated as a confluent culture for 48h. Cells were then switched to AIM for further 48 h and treated with budesonide, halcinonide and clobetasol while unstimulated cells were kept in pre-adipocyte expansion medium. The bar plot represent Gli1 expression determined by quantitative PCR. (n = 3) ± SEM. SAG and Itraconazole were used as positive and negative control for Gli1 induction respectively. Tubulin was used as housekeeping gene. SEM is reported. Statistical significance tested by one-way ANOVA (*p≤0.05, **p ≤0.01, ****p≤ 0.0001, ns: not significant). (b) Log2CPM expression levels for Gli 1, 2 and 3 genes. Expression data were extrapolated from the RNAseq experiment in which FAPs were treated with vehicle (DMSO) or 5 μM Budesonide for 24 hours. A post-hoc t-test has been applied and defined as *p≤0.05, **p ≤0.01, ****p≤ 0.0001, ns: not significant. (n = 3)

**Fig. S4 Budesonide treatment affects cell proliferation in *mdx* but not WT FAPs.** WT and *mdx* FAPs were isolated by the standard procedure and plated in fGM. 24 hours after seeding, cells were treated for further 6 days with 1 μM or 5 μM of budesonide and the effect of budesonide on apoptosis was analyzed at T0 = treatment, T1 = 48h upon treatment and T3 = 144h upon treatment. (a) Schematic representation of the experiment. Immunofluorescence microphotographs showing FAPs labelled using Ki-67 antibody (red) and with Hoechst 33342 for nuclei (grey) for WT (c) or *mdx* (d) cells. Scale bar: 100 μm. (e, f) Bar plots representing the percentage of Ki67-positive cells per field and total nuclei number per field for WT and *mdx* FAPs respectively. Data are represented as average ± SEM (n = 3), and statistical significance was evaluated using the one-way ANOVA test (*p≤0.05, ***p ≤0.001, ns: not significant).

**Fig. S5 Budesonide promotes apoptosis of WT and *mdx* FAPs.** WT and *mdx* FAPs were isolated by the standard procedure and plated in fGM. 24 hours after seeding, cells were treated for further 6 days with 1 μM or 5 μM of budesonide and its effect on cell proliferation and apoptosis was analyzed at T0 = treatment, T1 = 48h upon treatment and T3 = 144h upon treatment. (a) Schematic representation of the experiment. (b-d) Immunofluorescence microphotographs show apoptotic cells detected by TUNEL staining (red) counterstained with Hoechst 33342 for nuclei (gray). Scale bar: 100 μm. White arrows within the insets indicate apoptotic spots. Scale bar: 50 μm. DNase treatment was used as positive control for TUNEL staining. Bar plots showing the ratio between Tunel^+^ fragment and nuclei per field and the number of nuclei per field for WT (e) and *mdx* (f) FAPs. Data are represented as average ± SEM (n = 3), and statistical significance was evaluated using the one-way ANOVA test (*p≤0.05, ***p ≤0.001, ****p ≤0.001, ns: not significant).

**Fig. S6. Budesonide treatment promotes terminal differentiation of *mdx* and WT satellite cells. (**a, b) SCs were isolated from muscles of *mdx* mice as CD45-/CD31-/ITGA7+ cells and plated in sGM. 48 hours after plating, cells were treated with two concentrations of budesonide (1 and 5 μM) for 5 additional days. Early myogenic differentiation was assessed by immunostaining with antibodies against Pax7 (a) and MyoD (b). Nuclei were counterstained with Hoechst 33342. (c) Schematic representation of the experiment reported in a and b. Charts showing the percentages of Pax7 (d) and MyoD (e) positive cells in the experiment in panels a and b. Information is provided as lines to better show the temporal changes of the myogenic markers. The values are mean of two independent experiments ± SEM. Statistical significance was evaluated using two-way ANOVA (**p≤0.01). Scale bar: 100 μm. SCs were isolated from muscles of C57BL/6 mice (WT) as CD31-/CD45-/a7Integrin+ cells and plated in sGM (DMEM, 20% FBS, 10% horse serum, 1% Chicken Embryo Extract). 48 hours after plating, cells were treated with three concentrations of budesonide or dexamethasone (0.1, 1 and 5 μM) for 5 additional days. Myogenic differentiation was assessed by immunostaining with antibodies against Myosin Heavy Chain (MyHC) while nuclei were stained with Hoechst 33342. The bar plots show the fusion index (f), the percentage of the field area covered by myotubes (g) and the number of nuclei per field for the experiments (h). Data are represented as average ± SEM (n = 2). Statistical significance was evaluated using one-way ANOVA (*p≤0.05, **p≤0.01, ns: not significant).

**Fig. S7 RU-486 suppresses the glucocorticoid-induced myogenic differentiation of C2C12 myoblast.** (a) Immunofluorescence microphotographs of C2C12 labelled with an antibody against MyHC (green) after 7 days of culture in cGM. 24 hours after seeding, cells were treated with budesonide or dexamethasone at different concentrations alone or in combination with RU-486 (2μM) for 6 additional days. Nuclei are stained with Hoechst 33342 (grey). (b, c) Bar plots showing the fusion index for the experiment reported in A. (n = 4) ± SEM, Statistical significance has been evaluated using one-way ANOVA (*p≤0.05, **p ≤0.01, ***p≤0.001, ****p≤ 0.0001, ns: not significant). Scale bar: 100 μm.

**Fig. S8 Treatment with IBMX or forskolin affect nuclei number or adipogenic differentiation of *mdx* FAPs.** *mdx* FAPs isolated by the standard procedure and plated in GM 20% FBS were supplemented with 0.5 mM IBMX 48 h after their seeding and cultured for further 5 days. (a) The microphotographs show nuclei stained with Hoechst 33342. Scale bar: 100 μm (b) The bar plot indicates the average number of nuclei per field. Scale bar: 100 μm. (c) *mdx* FAPs were isolated by the standard procedure and plated in fGM. 24h upon seeding, cells were treated with increasing concentrations of forskolin in presence or absence of budesonide 5 μM for further 6 days. Adipogenic differentiation was assessed using Oil red o (ORO) staining to reveal adipocytes and Hoechst 33342 to reveal nuclei. The plot shows the percentage of adipocytes for a specific range of total corrected cellular fluorescence (TCCF) intensity value (expressed in arbitrary units AU) for control cells or cells treated with forskolin 10 μM alone or in combination with budesonide 5 μM. (d) Box plot showing the nuclei number per field. Box plots show median and interquartile range with whiskers extended to minimum and maximum values (n = 3). Statistical significance has been evaluated using one-way ANOVA (*p≤0.05, **p ≤0.01, ns: not significant).

**Fig. S9.** **Transcriptome analysis of *mdx* FAPs treated with budesonide.** (a) Multi-scatter plot of budesonide treated and control profiles, which highlights higher correlation between budesonide treated samples (mean correlation coefficient 0.9405) and control samples (mean correlation coefficient 0.963) in comparison with budesonide-treaded VS control samples (mean correlation coefficient 0.914). (b) The principal component analysis reveals a separation of budesonide-treated (blue area) and control samples (read area). (c) Density scatter plot of the expression in CPM (counts per million) of all genes of budesonide and control samples. Black dots represent genes whose enrichment id statistically significant genes. (d) Heatmap and hierarchical clustering of the significant (FDR < 0.05) differentially expressed genes in the two conditions. (e) Table showing the top 10 up-regulated (left) and down-regulated (right) genes in the differential expression analysis. (f) Heatmap showing Pearson correlation values between samples. Samples are clustered according to the grouping (budesonide-treated VS control samples). (n = 3).

**Fig. S10 Full-length gel of Fig. 1c**

**Fig. S11 Full-length gel of Fig. 7c**

**Fig. S12 Full-length gel of Fig. 7g**

**Table S1.** The table shows nuclei count and adipogenic differentiation of *mdx* FAPs for the 8 GCs of the Prestwick library displaying antiadipogenic activity. Values are expressed as percentage compared to DMSO treated cells.

**Table S2.** The table shows the list of significantly modulated genes in *mdx* FAPs treated with 5 μM budesonide for 24 hours.

**Fig. S1**

**
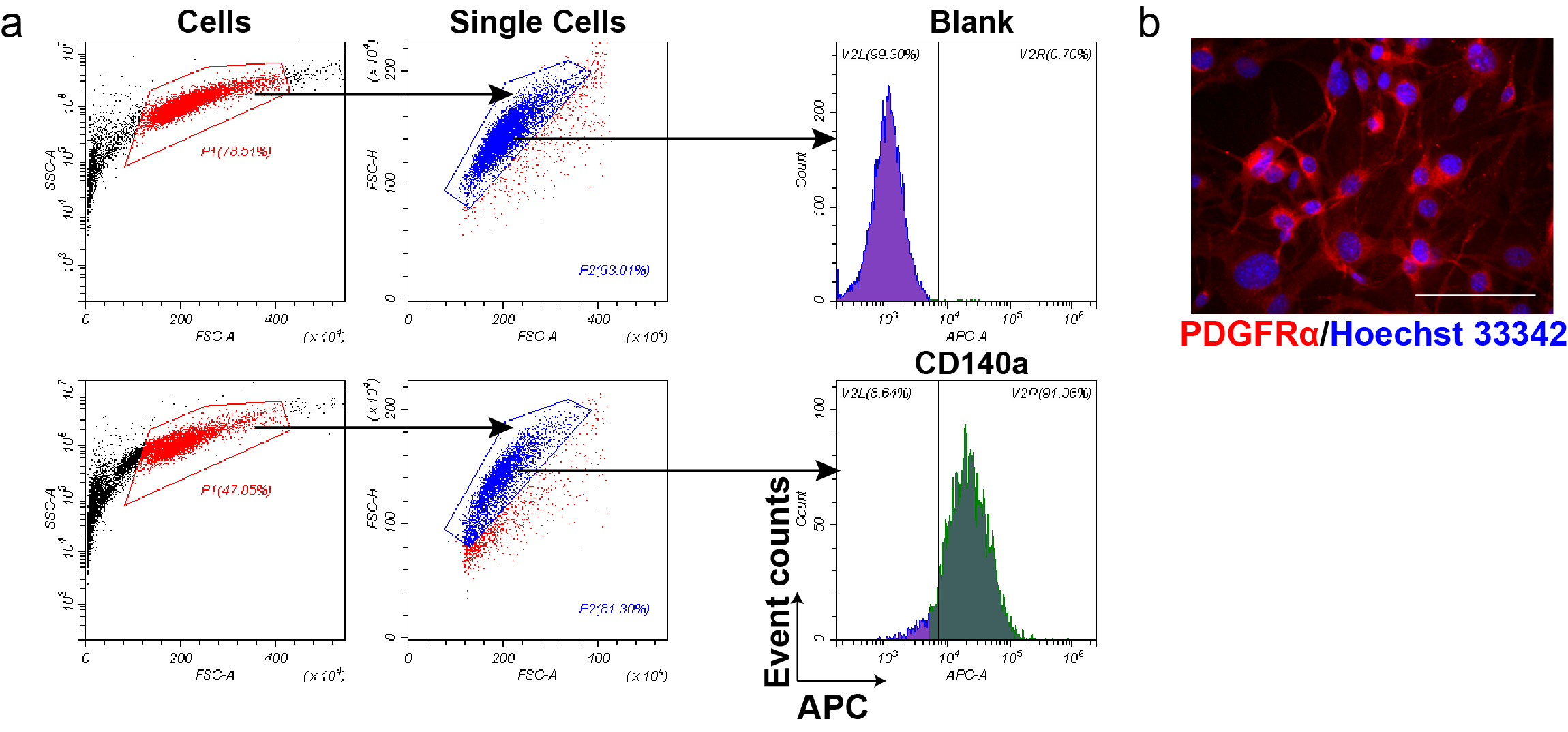
**

**Fig. S2**

**
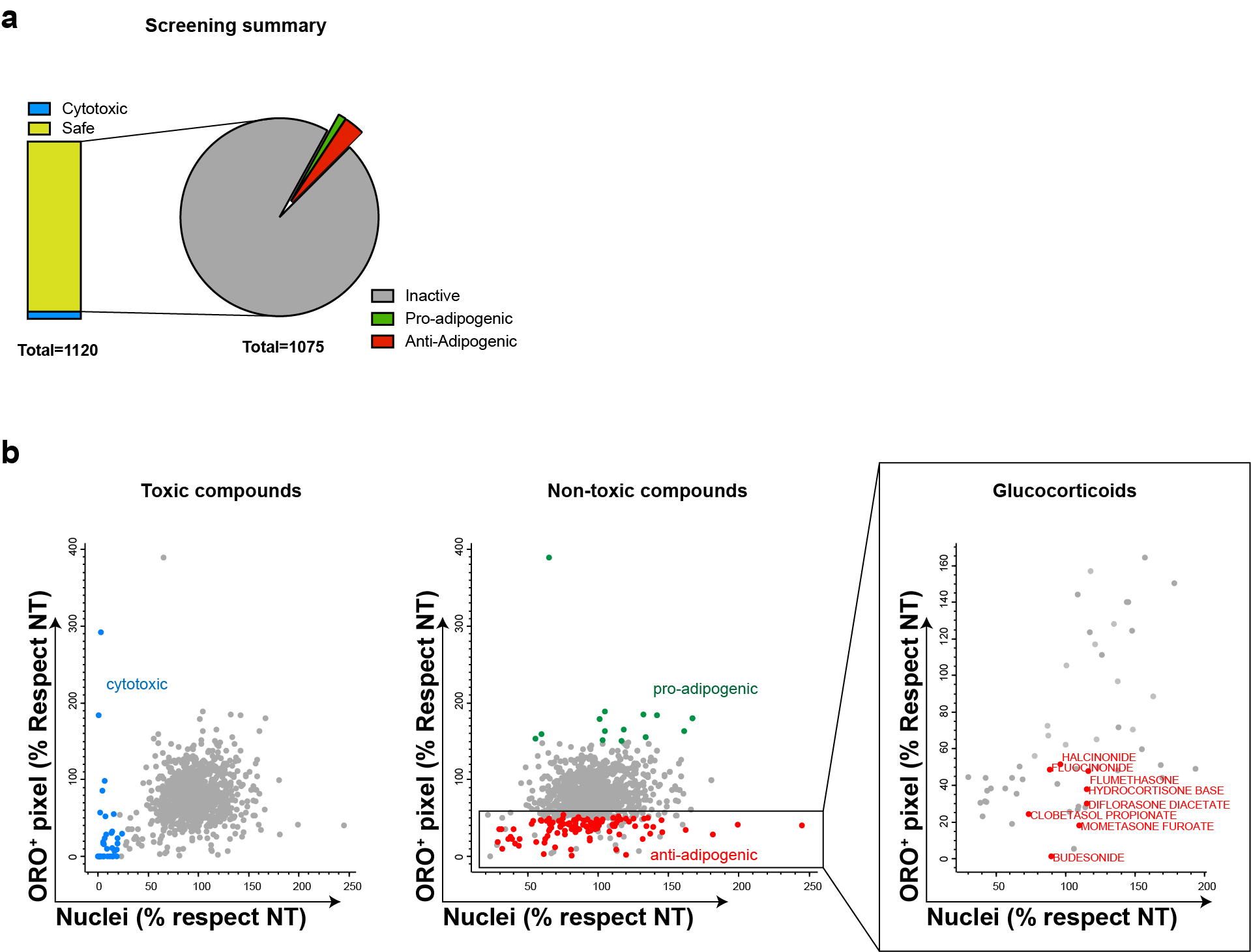
**

**Fig. S3**

**
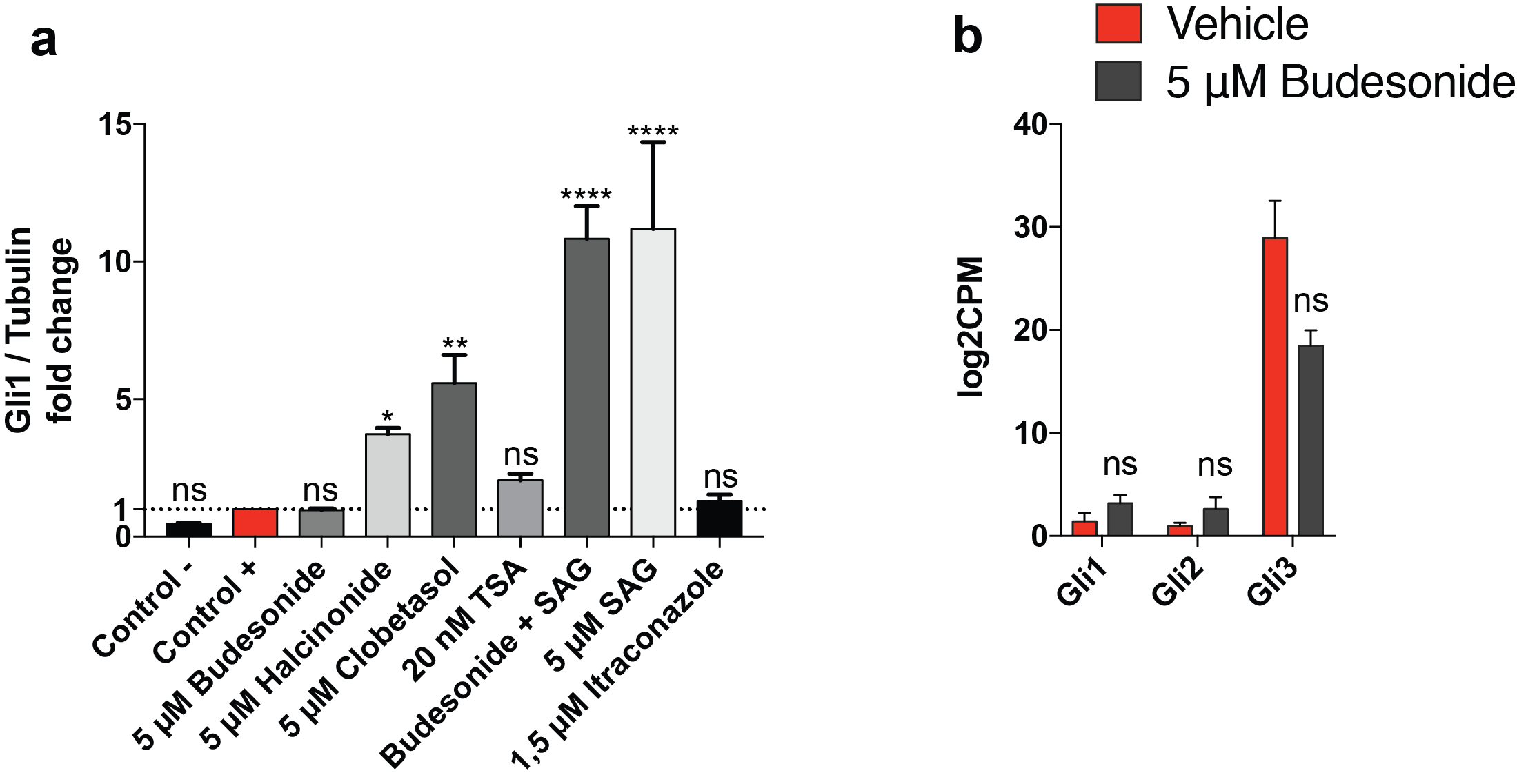
**

**Fig. S4**

**
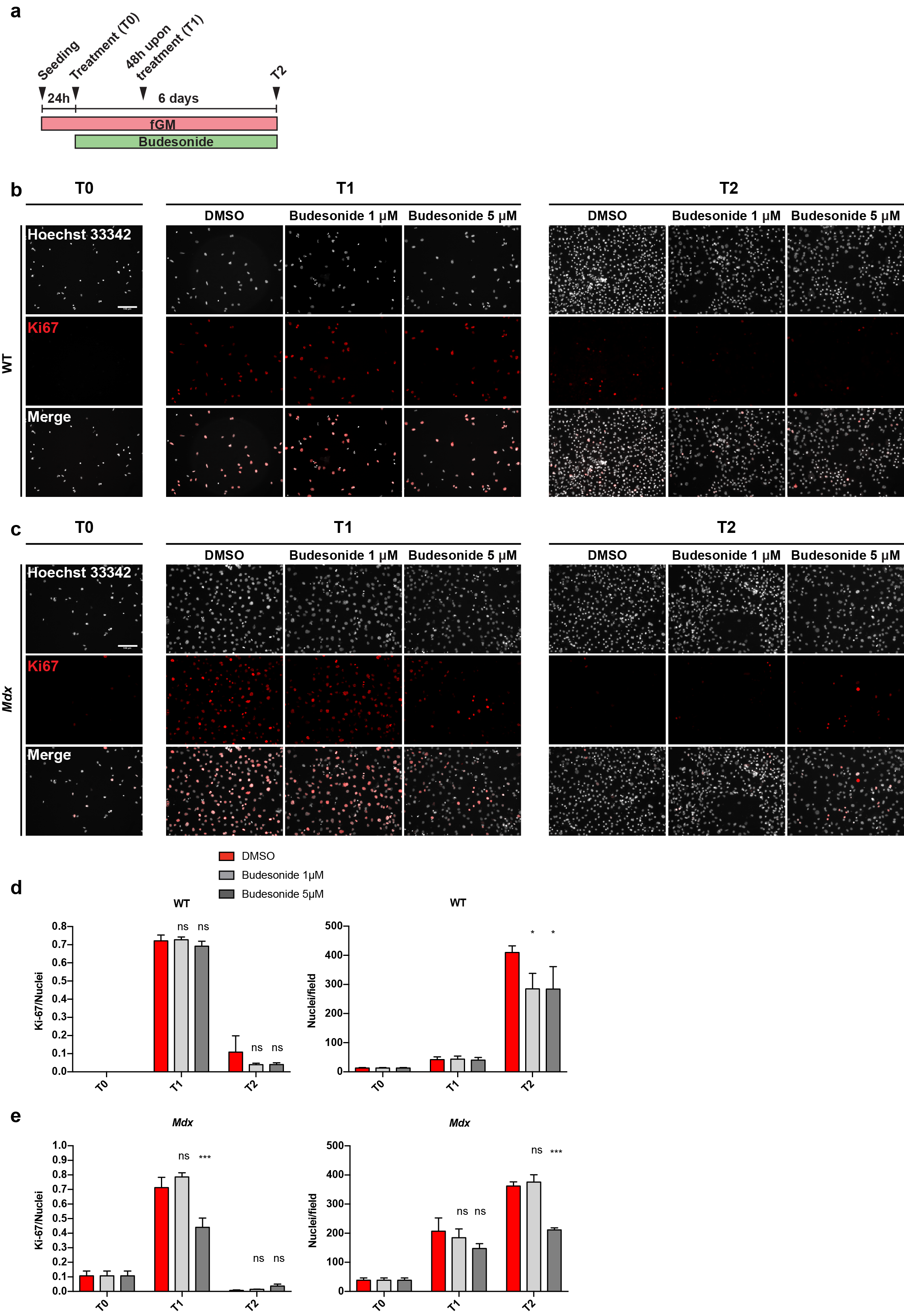
**

**Fig. S5**

**
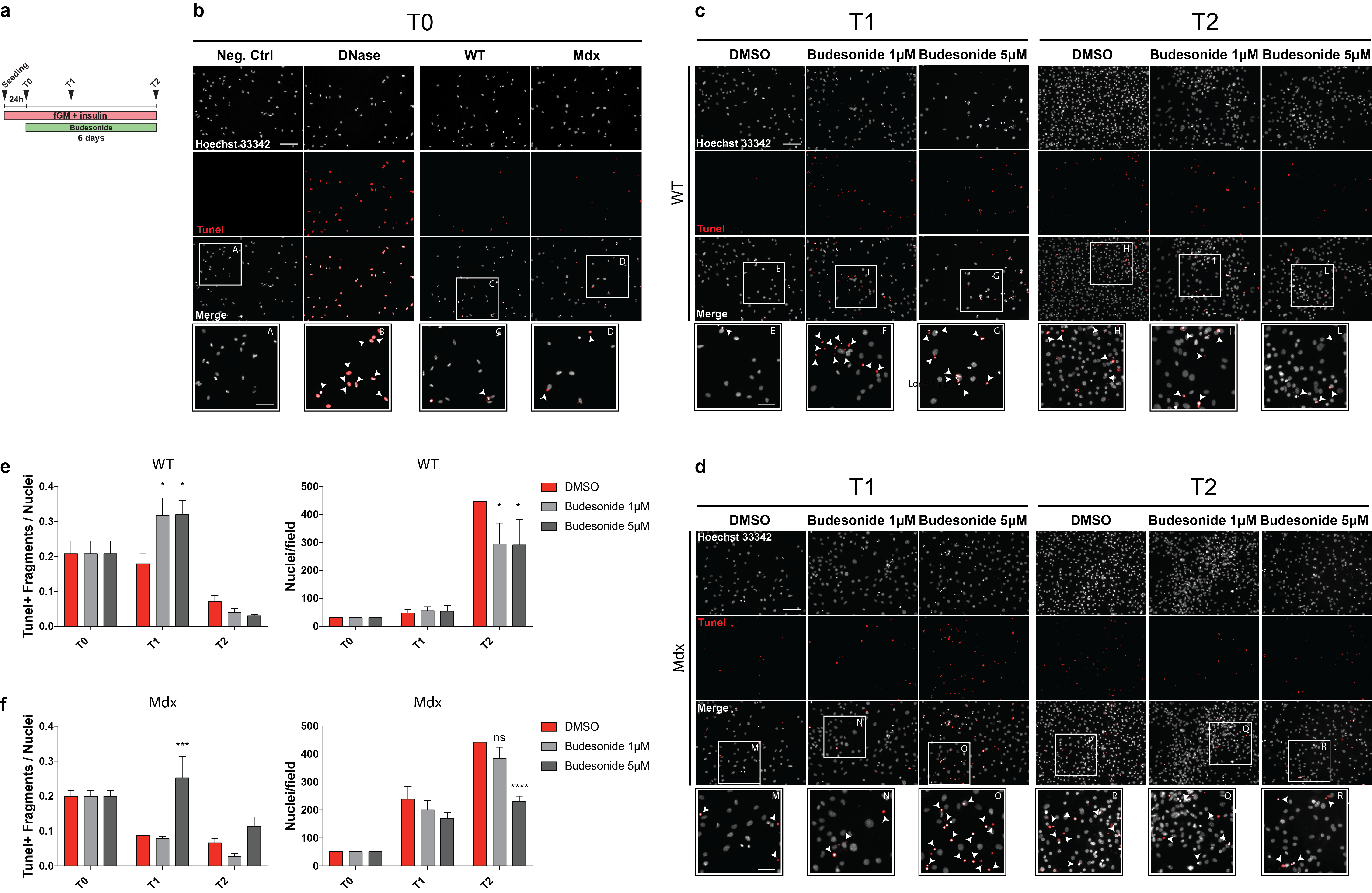
**

**Fig. S6**

**
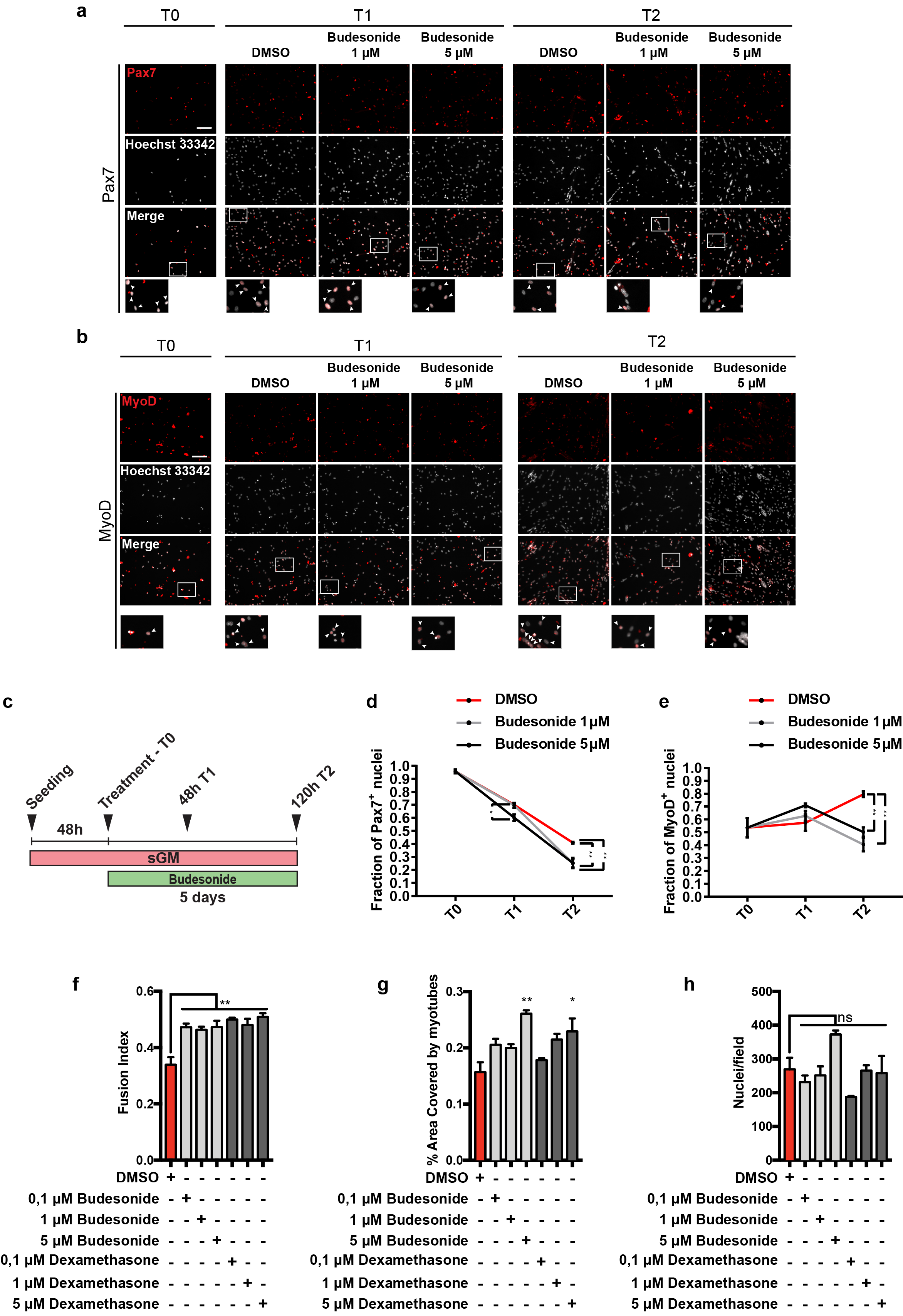
**

**Fig. S7**

**
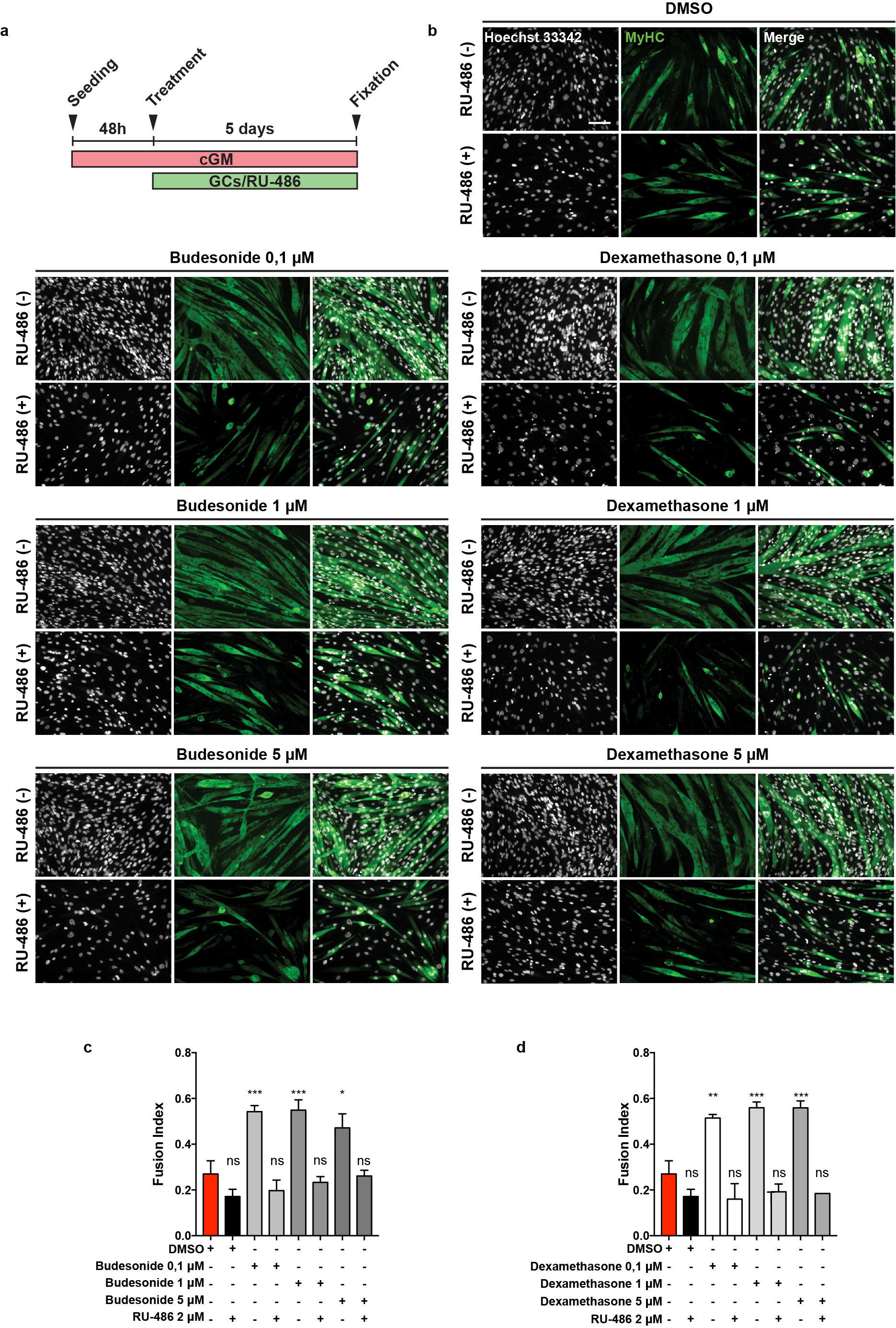
**

**Fig. S8**


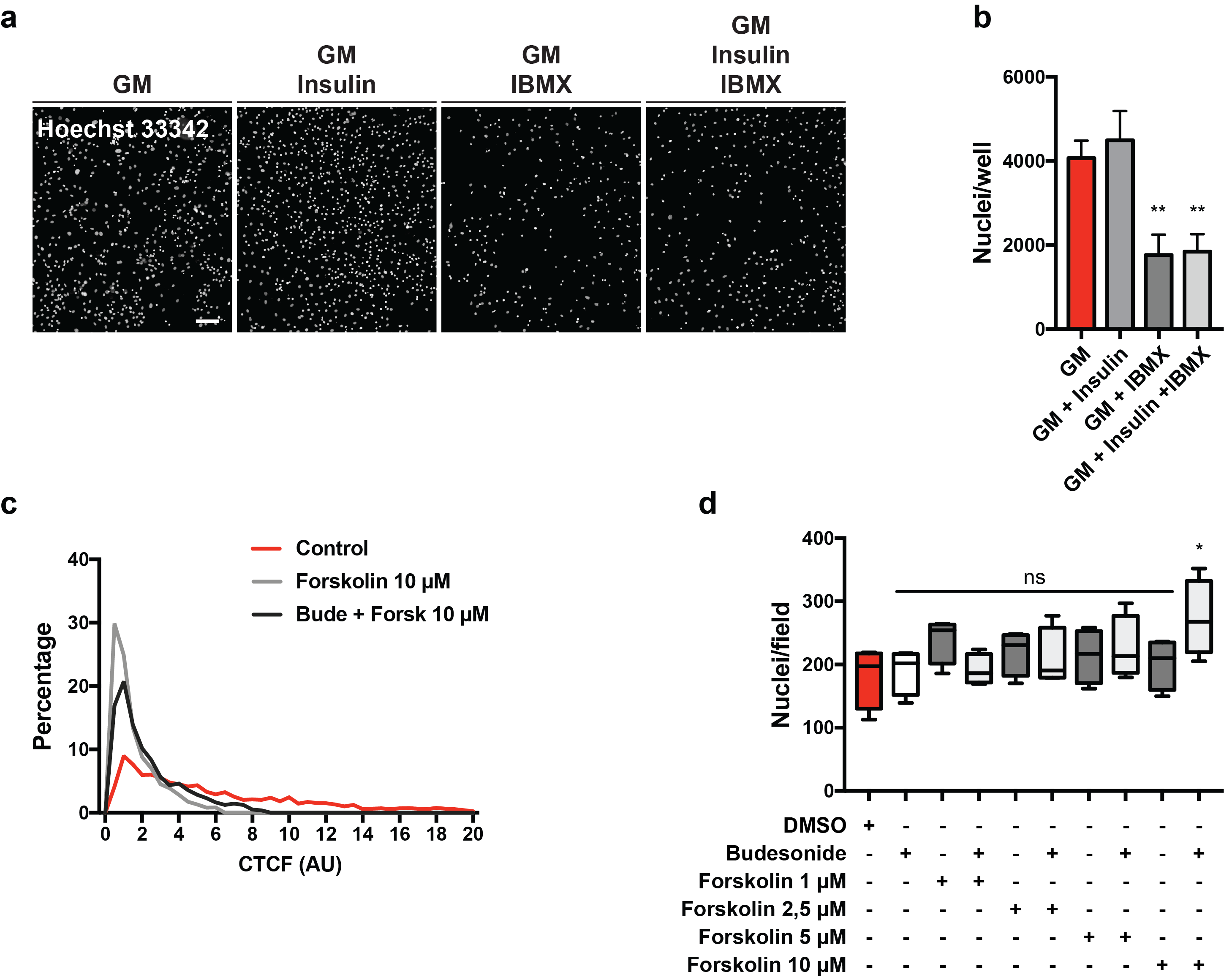


**Fig. S9**


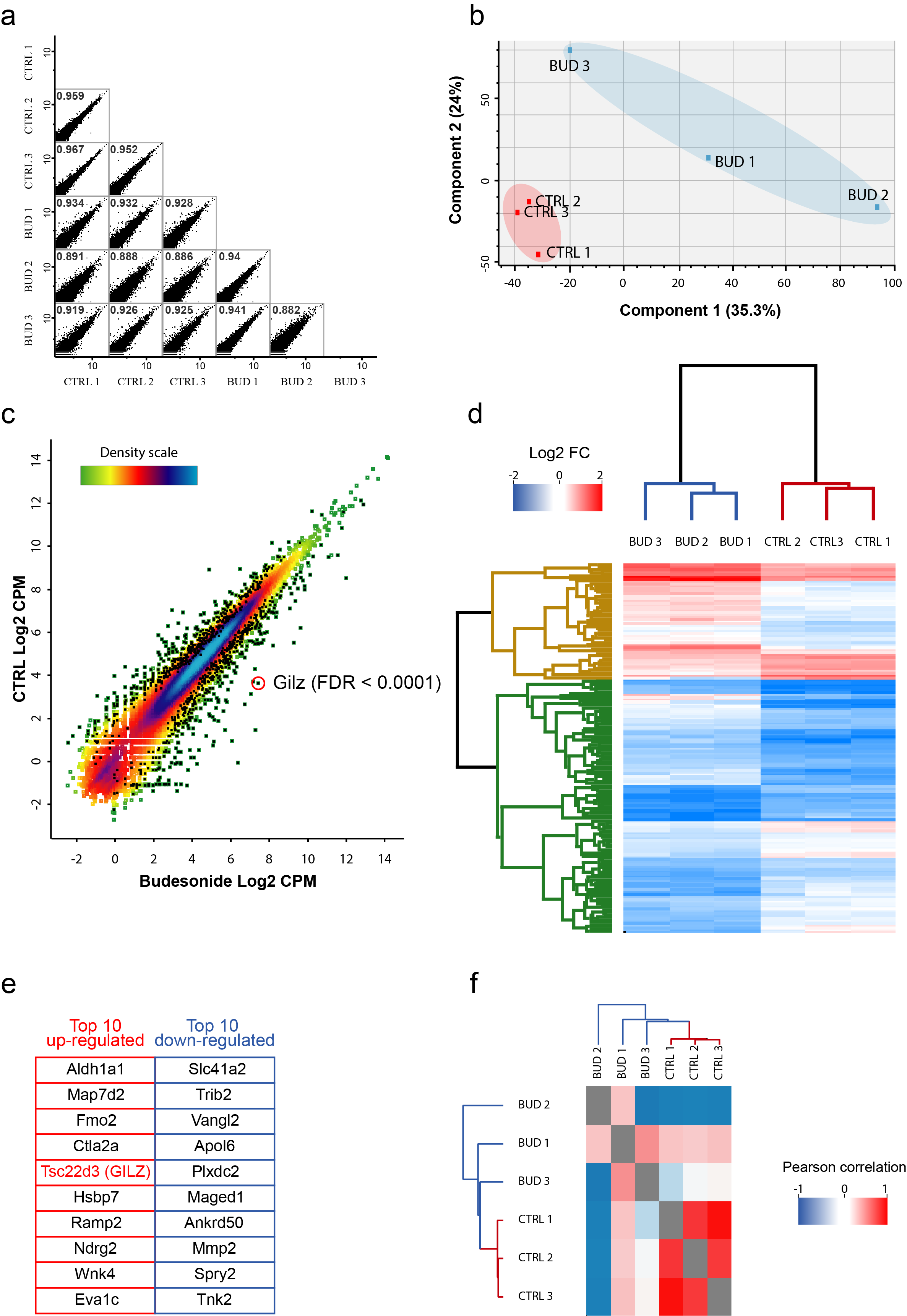


**Fig. S10**

**
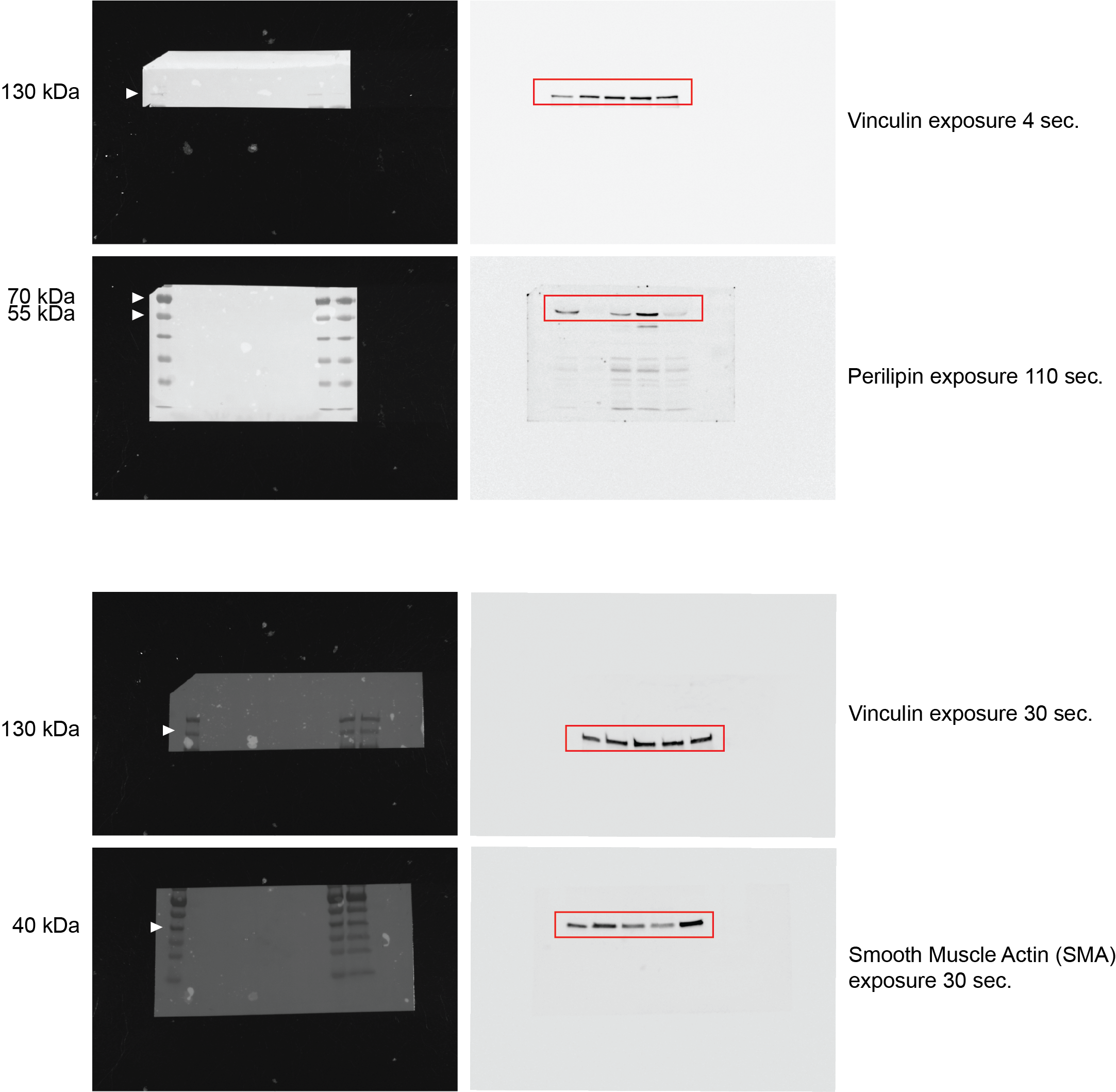
**

**Fig. S11**

**
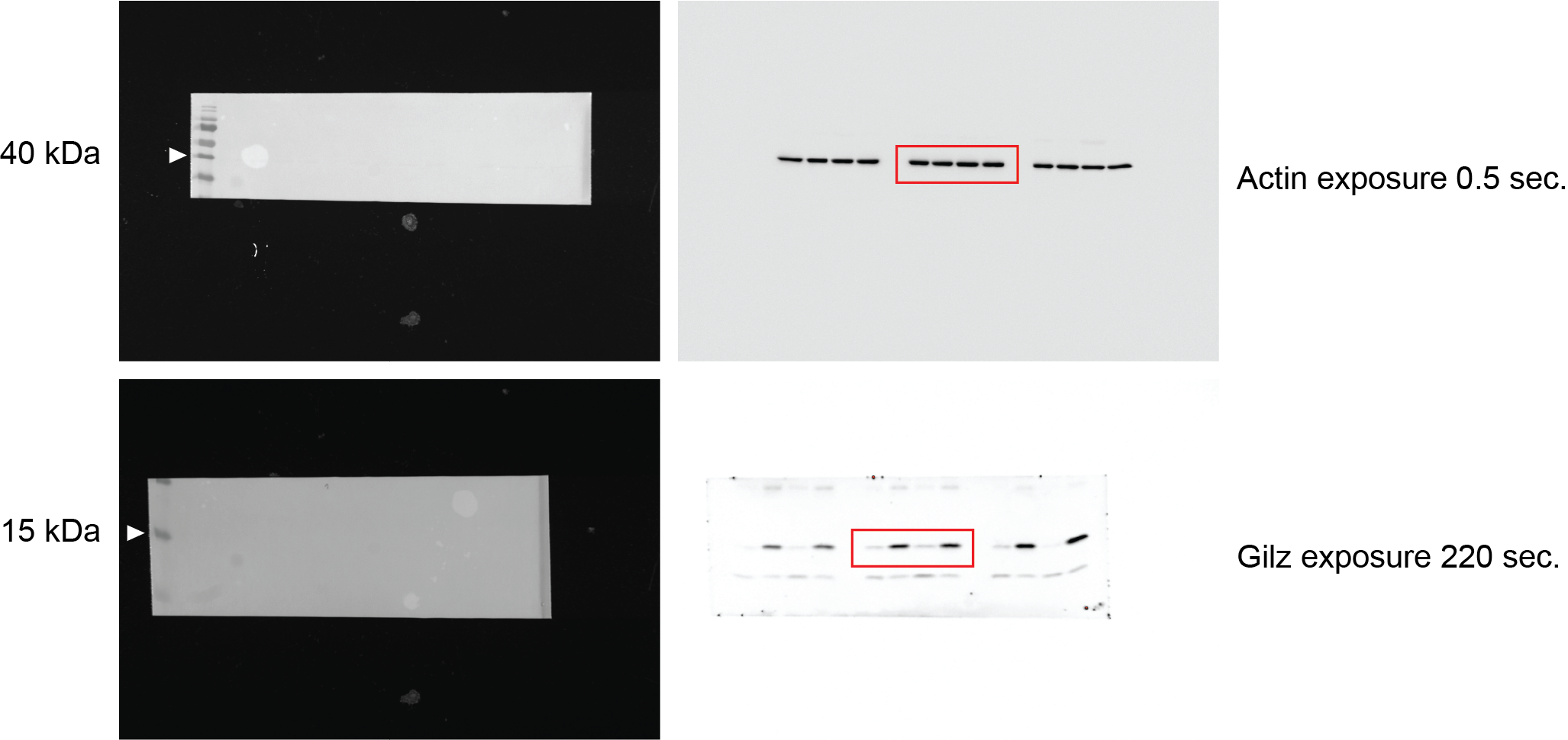
**

**Fig. S12**

**
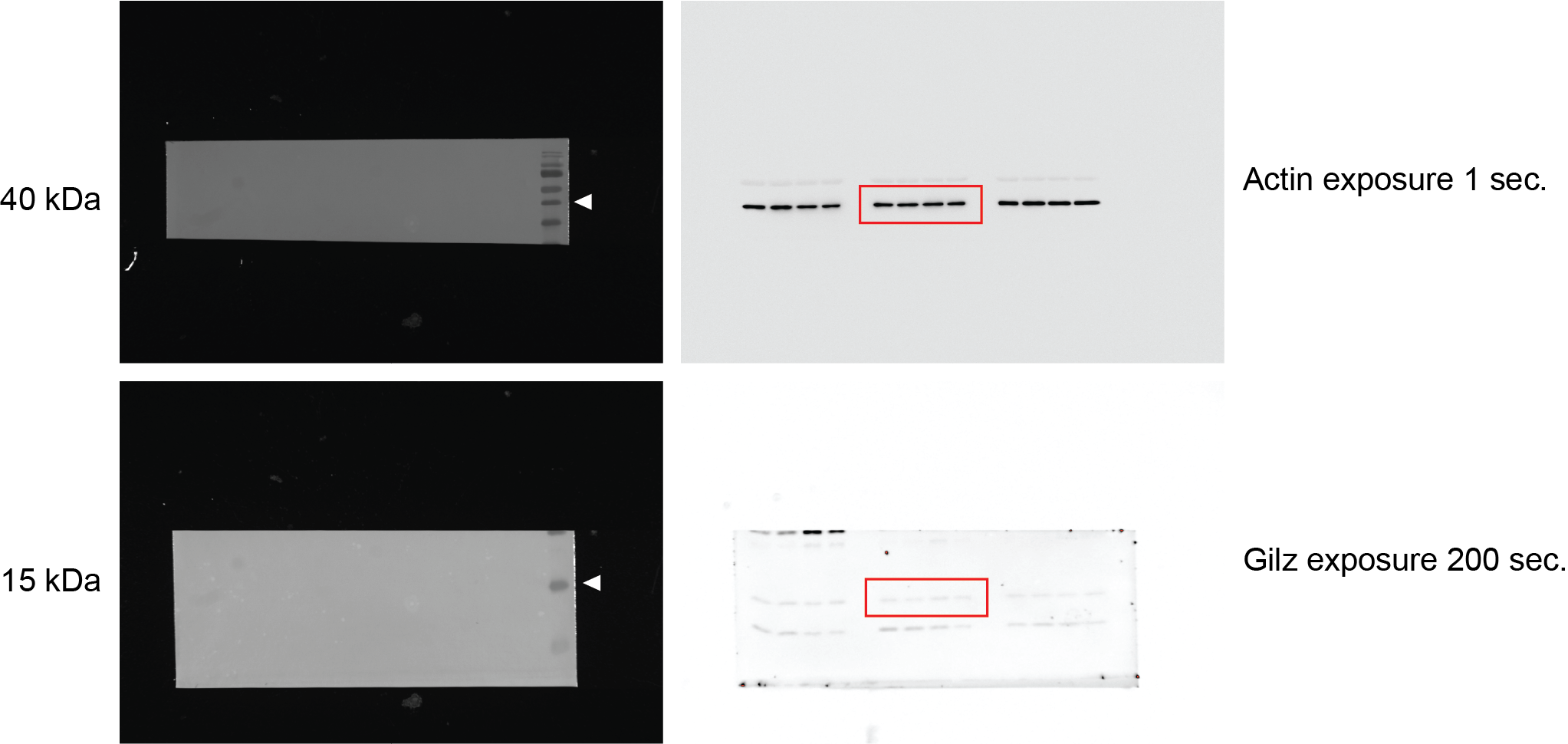
**

**Table S1**

|  | **Nuclei count** | **Adipogenic**  **differentiation** |
| --- | --- | --- |
| **Drug Name** | % compared to DMSO | % compared to DMSO |
| MOMETASONE FUROATE | *109,8* | *18,0* |
| HYDROCORTISONE BASE | *115,1* | *37,9* |
| DIFLORASONE DIACETATE | *115,1* | *30,2* |
| FLUMETHASONE | *116,4* | *47,9* |
| HALCINONIDE | *96,4* | *51,6* |
| CLOBETASOL PROPIONATE | *73,2* | *24,3* |
| BUDESONIDE | *89,9* | *1,2* |
| FLUOCINONIDE | 88,5 | 48,5 |

**Table S2**

| **Gene_Symbol** | **Control_3** | **Control_1** | **Control_2** | **Budsonide_3** | **Budesonide_1** | **Budsonide_2** | **Student's t-test Significant B_C** | **-Log Student's t-test p-value B_C** | **Student's t-test q-value B_C** | **log2 Fold Change (Bud/Con)** |
| --- | --- | --- | --- | --- | --- | --- | --- | --- | --- | --- |
| Ccl9 | 7.26 | 7.04 | 5.65 | 2.96 | 2.70 | 3.11 | + | 2.70 | 0.01 | -3.73 |
| Pfkfb3 | 5.73 | 5.68 | 5.18 | 2.96 | 2.43 | 0.53 | + | 2.02 | 0.05 | -3.55 |
| Cxcl5 | 9.17 | 8.92 | 8.37 | 6.52 | 4.88 | 4.61 | + | 2.25 | 0.04 | -3.48 |
| Gfra1 | 6.07 | 5.87 | 4.16 | 1.55 | 2.23 | 2.40 | + | 2.13 | 0.04 | -3.31 |
| Il1rl1 | 6.21 | 5.54 | 5.25 | 2.55 | 1.70 | 2.84 | + | 2.75 | 0.01 | -3.31 |
| Angptl4 | 6.62 | 6.43 | 6.41 | 3.29 | 3.05 | 3.46 | + | 4.72 | 0.00 | -3.22 |
| Alpl | 6.28 | 6.76 | 5.39 | 3.55 | 2.61 | 2.74 | + | 2.51 | 0.02 | -3.17 |
| Pde4b | 4.83 | 4.24 | 3.27 | 1.55 | 1.11 | 0.53 | + | 2.31 | 0.03 | -3.05 |
| Tgfbi | 7.12 | 6.93 | 5.98 | 4.05 | 3.53 | 3.52 | + | 2.79 | 0.01 | -2.97 |
| Col23a1 | 3.46 | 3.77 | 3.12 | 0.96 | 0.11 | 1.26 | + | 2.61 | 0.02 | -2.67 |
| Arsi | 5.71 | 5.55 | 4.80 | 2.55 | 3.11 | 2.64 | + | 2.85 | 0.01 | -2.59 |
| Aldh1a2 | 5.88 | 5.35 | 5.16 | 3.77 | 2.23 | 2.64 | + | 2.15 | 0.05 | -2.59 |
| Elfn1 | 4.16 | 3.30 | 3.70 | 1.55 | 1.33 | 0.53 | + | 2.54 | 0.02 | -2.58 |
| Apln | 5.87 | 5.23 | 4.75 | 2.77 | 2.33 | 3.11 | + | 2.53 | 0.02 | -2.55 |
| Piezo2 | 5.54 | 5.75 | 5.79 | 2.77 | 3.23 | 3.46 | + | 3.52 | 0.01 | -2.54 |
| Lmx1a | 3.57 | 3.12 | 3.03 | 0.96 | 0.85 | 0.53 | + | 3.50 | 0.02 | -2.46 |
| Cpxm1 | 6.19 | 6.37 | 6.18 | 2.96 | 4.17 | 4.29 | + | 2.33 | 0.04 | -2.44 |
| Mest | 6.13 | 5.89 | 5.82 | 3.66 | 3.95 | 2.94 | + | 2.81 | 0.01 | -2.43 |
| Arl4c | 4.96 | 4.64 | 5.44 | 2.96 | 2.23 | 2.64 | + | 2.81 | 0.01 | -2.40 |
| Arhgef19 | 4.04 | 3.92 | 3.75 | 2.29 | 1.53 | 0.93 | + | 2.36 | 0.04 | -2.32 |
| Itga11 | 5.10 | 5.34 | 4.95 | 2.55 | 2.77 | 3.11 | + | 3.51 | 0.01 | -2.32 |
| Ptn | 6.03 | 6.90 | 7.18 | 4.13 | 4.61 | 4.66 | + | 2.36 | 0.04 | -2.24 |
| Angptl2 | 6.83 | 7.09 | 6.78 | 5.42 | 4.33 | 4.43 | + | 2.41 | 0.04 | -2.17 |
| Kif26b | 5.63 | 5.93 | 5.85 | 3.77 | 3.61 | 3.52 | + | 4.31 | 0.02 | -2.17 |
| Epha4 | 4.26 | 4.77 | 4.85 | 2.29 | 3.05 | 2.40 | + | 2.62 | 0.02 | -2.05 |
| Ptgfr | 6.87 | 7.05 | 6.28 | 5.21 | 4.33 | 4.61 | + | 2.35 | 0.04 | -2.01 |
| Chst11 | 4.12 | 3.55 | 3.99 | 2.29 | 1.99 | 1.52 | + | 2.65 | 0.02 | -1.96 |
| St3gal5 | 6.23 | 6.15 | 6.35 | 4.96 | 3.70 | 4.22 | + | 2.19 | 0.05 | -1.95 |
| Plppr3 | 5.87 | 5.73 | 5.23 | 3.66 | 3.43 | 4.02 | + | 2.74 | 0.02 | -1.90 |
| Tmem26 | 5.06 | 5.03 | 4.53 | 3.42 | 2.77 | 2.74 | + | 2.60 | 0.03 | -1.89 |
| Rnf150 | 5.68 | 5.54 | 5.08 | 3.66 | 3.38 | 3.58 | + | 3.15 | 0.01 | -1.89 |
| Il4ra | 10.18 | 10.21 | 10.14 | 8.62 | 8.08 | 8.15 | + | 3.43 | 0.01 | -1.89 |
| Ptger4 | 5.88 | 5.53 | 5.12 | 3.55 | 3.66 | 3.74 | + | 2.90 | 0.01 | -1.86 |
| Megf6 | 3.63 | 4.12 | 3.90 | 1.96 | 2.23 | 1.94 | + | 3.38 | 0.01 | -1.84 |
| Gpr39 | 5.92 | 5.87 | 5.75 | 3.87 | 4.28 | 4.11 | + | 3.79 | 0.01 | -1.76 |
| Maz | 3.04 | 2.30 | 2.85 | 0.96 | 1.11 | 0.93 | + | 2.78 | 0.02 | -1.73 |
| Tmem138 | 3.12 | 3.41 | 3.19 | 1.96 | 1.11 | 1.52 | + | 2.55 | 0.03 | -1.71 |
| Pxylp1 | 6.79 | 7.32 | 6.91 | 5.72 | 5.20 | 5.00 | + | 2.50 | 0.04 | -1.70 |
| Chsy1 | 9.02 | 8.60 | 8.26 | 7.09 | 7.06 | 6.66 | + | 2.54 | 0.03 | -1.69 |
| Podnl1 | 5.50 | 6.03 | 5.91 | 3.77 | 4.38 | 4.26 | + | 2.61 | 0.03 | -1.68 |
| Igf1 | 8.24 | 8.22 | 8.34 | 6.71 | 6.96 | 6.19 | + | 2.70 | 0.02 | -1.65 |
| Emilin1 | 6.26 | 6.11 | 6.05 | 4.82 | 4.77 | 3.94 | + | 2.29 | 0.05 | -1.63 |
| Mme | 5.80 | 6.10 | 6.30 | 4.21 | 4.61 | 4.55 | + | 2.96 | 0.02 | -1.61 |
| Abtb2 | 2.68 | 3.18 | 3.12 | 1.55 | 1.70 | 0.93 | + | 2.32 | 0.05 | -1.60 |
| Aqp1 | 5.73 | 6.28 | 5.45 | 3.96 | 4.38 | 4.40 | + | 2.30 | 0.05 | -1.57 |
| Gab1 | 3.12 | 2.92 | 3.19 | 1.55 | 1.33 | 1.74 | + | 3.36 | 0.01 | -1.54 |
| Tmem2 | 6.71 | 6.93 | 6.97 | 5.64 | 5.33 | 5.07 | + | 2.93 | 0.02 | -1.52 |
| Tmtc4 | 4.16 | 4.24 | 4.03 | 2.77 | 2.77 | 2.40 | + | 3.37 | 0.01 | -1.50 |
| Prickle2 | 5.86 | 6.10 | 5.67 | 4.55 | 4.20 | 4.40 | + | 3.14 | 0.01 | -1.50 |
| Cdc6 | 2.57 | 2.60 | 2.27 | 0.96 | 1.11 | 0.93 | + | 3.61 | 0.01 | -1.48 |
| C1qtnf6 | 7.35 | 7.50 | 7.16 | 5.75 | 6.08 | 5.77 | + | 3.25 | 0.01 | -1.47 |
| Col16a1 | 8.63 | 8.71 | 8.84 | 7.50 | 7.30 | 7.07 | + | 3.31 | 0.01 | -1.44 |
| Rai2 | 6.24 | 6.35 | 6.28 | 5.17 | 4.57 | 4.96 | + | 2.82 | 0.03 | -1.39 |
| Nfatc1 | 4.19 | 3.99 | 3.95 | 2.55 | 2.99 | 2.52 | + | 2.89 | 0.02 | -1.36 |
| Igsf10 | 4.83 | 4.69 | 4.80 | 3.29 | 3.66 | 3.40 | + | 3.45 | 0.01 | -1.33 |
| Pcnx3 | 6.89 | 7.18 | 6.76 | 5.75 | 5.75 | 5.40 | + | 2.81 | 0.03 | -1.31 |
| Fam19a5 | 3.12 | 2.99 | 3.19 | 1.96 | 1.70 | 1.74 | + | 3.68 | 0.02 | -1.30 |
| Nxn | 8.58 | 8.67 | 8.46 | 7.09 | 7.14 | 7.60 | + | 2.77 | 0.03 | -1.29 |
| Wwp2 | 7.07 | 7.20 | 6.71 | 5.96 | 5.72 | 5.52 | + | 2.54 | 0.05 | -1.26 |
| Dpysl3 | 9.29 | 9.13 | 8.71 | 7.83 | 7.69 | 7.83 | + | 2.68 | 0.04 | -1.26 |
| Rgs4 | 4.33 | 4.43 | 4.50 | 3.13 | 3.53 | 2.84 | + | 2.45 | 0.05 | -1.25 |
| Adamts4 | 6.05 | 5.98 | 5.84 | 4.61 | 4.72 | 4.82 | + | 3.86 | 0.02 | -1.24 |
| B4galt3 | 5.25 | 5.42 | 5.27 | 3.77 | 4.25 | 4.22 | + | 2.76 | 0.04 | -1.23 |
| Sncaip | 4.00 | 4.35 | 4.27 | 2.96 | 3.28 | 2.74 | + | 2.52 | 0.05 | -1.21 |
| Spcs3 | 2.46 | 2.41 | 2.53 | 1.55 | 1.11 | 1.26 | + | 3.00 | 0.03 | -1.16 |
| Tln2 | 8.38 | 8.62 | 8.24 | 7.23 | 7.51 | 7.22 | + | 2.77 | 0.04 | -1.09 |
| Hcfc2 | 4.87 | 4.67 | 4.65 | 3.77 | 3.61 | 3.58 | + | 3.49 | 0.02 | -1.07 |
| Gm28151 | 1.68 | 1.92 | 1.94 | 0.96 | 0.85 | 0.53 | + | 2.62 | 0.05 | -1.07 |
| Tmem268 | 4.46 | 4.35 | 4.70 | 3.66 | 3.43 | 3.40 | + | 2.79 | 0.05 | -1.01 |
| Tnk2 | 4.36 | 4.41 | 4.37 | 3.29 | 3.33 | 3.52 | + | 3.78 | 0.01 | -1.00 |
| Spry2 | 6.19 | 5.97 | 6.03 | 5.25 | 4.97 | 5.00 | + | 3.08 | 0.03 | -0.99 |
| Mmp2 | 8.34 | 8.23 | 8.33 | 7.38 | 7.33 | 7.21 | + | 4.06 | 0.01 | -0.99 |
| Ankrd50 | 6.08 | 6.12 | 6.10 | 5.25 | 5.03 | 5.11 | + | 3.94 | 0.01 | -0.97 |
| Maged1 | 8.65 | 8.68 | 8.93 | 7.83 | 7.75 | 7.88 | + | 3.18 | 0.03 | -0.93 |
| Plxdc2 | 7.79 | 7.51 | 7.76 | 6.72 | 6.74 | 6.92 | + | 2.92 | 0.04 | -0.90 |
| Apol6 | 6.14 | 6.07 | 5.91 | 5.29 | 5.13 | 5.13 | + | 3.26 | 0.03 | -0.86 |
| Vangl2 | 5.67 | 5.60 | 5.41 | 4.61 | 4.68 | 4.82 | + | 2.99 | 0.05 | -0.86 |
| Trib2 | 6.12 | 6.22 | 6.19 | 5.46 | 5.35 | 5.28 | + | 3.77 | 0.02 | -0.82 |
| Slc41a2 | 5.78 | 5.85 | 5.78 | 5.01 | 4.97 | 4.98 | + | 5.24 | 0.02 | -0.82 |
| Sipa1l1 | 5.54 | 5.68 | 5.60 | 6.37 | 6.35 | 6.40 | + | 4.32 | 0.02 | 0.76 |
| Six1 | 5.92 | 6.01 | 6.13 | 6.91 | 6.87 | 6.81 | + | 3.64 | 0.02 | 0.84 |
| Ezh1 | 5.59 | 5.78 | 5.71 | 6.56 | 6.51 | 6.59 | + | 3.81 | 0.02 | 0.86 |
| Slc7a6os | 3.92 | 3.64 | 3.95 | 4.77 | 4.72 | 4.82 | + | 3.12 | 0.04 | 0.93 |
| Cd34 | 6.16 | 6.32 | 6.47 | 7.34 | 7.10 | 7.34 | + | 2.85 | 0.05 | 0.95 |
| Scn2a | 3.40 | 3.30 | 3.59 | 4.36 | 4.28 | 4.52 | + | 3.00 | 0.04 | 0.96 |
| Zhx3 | 6.17 | 6.12 | 5.89 | 6.91 | 6.97 | 7.22 | + | 2.78 | 0.05 | 0.97 |
| Mxi1 | 4.52 | 4.55 | 4.83 | 5.64 | 5.68 | 5.82 | + | 3.17 | 0.02 | 1.08 |
| Rsph9 | 2.68 | 2.41 | 2.41 | 3.55 | 3.53 | 3.69 | + | 3.32 | 0.02 | 1.09 |
| Prdx6 | 8.67 | 8.55 | 8.26 | 9.46 | 9.68 | 9.65 | + | 2.85 | 0.04 | 1.10 |
| Thsd7a | 8.28 | 8.47 | 8.20 | 9.28 | 9.30 | 9.73 | + | 2.59 | 0.05 | 1.12 |
| Pxmp2 | 2.87 | 2.85 | 3.12 | 4.29 | 3.85 | 4.07 | + | 2.74 | 0.04 | 1.12 |
| Rhoj | 7.36 | 7.24 | 7.28 | 8.70 | 8.27 | 8.31 | + | 2.88 | 0.03 | 1.13 |
| C530043K16Rik | 5.14 | 5.27 | 5.29 | 6.17 | 6.26 | 6.69 | + | 2.62 | 0.05 | 1.14 |
| Sox5 | 3.52 | 2.92 | 3.12 | 4.29 | 4.38 | 4.40 | + | 2.56 | 0.05 | 1.17 |
| Grina | 7.08 | 7.14 | 7.38 | 8.22 | 8.25 | 8.73 | + | 2.50 | 0.05 | 1.20 |
| Ssh2 | 6.25 | 6.19 | 6.20 | 7.11 | 7.47 | 7.66 | + | 2.76 | 0.04 | 1.20 |
| Ak3 | 4.94 | 4.79 | 4.65 | 6.30 | 5.76 | 5.94 | + | 2.59 | 0.05 | 1.21 |
| Rin3 | 2.04 | 2.51 | 2.27 | 3.66 | 3.23 | 3.58 | + | 2.52 | 0.05 | 1.22 |
| Nupr1 | 6.91 | 6.89 | 6.93 | 7.85 | 8.31 | 8.24 | + | 3.00 | 0.02 | 1.22 |
| Gm44834 | 3.04 | 3.30 | 2.85 | 4.13 | 4.23 | 4.66 | + | 2.44 | 0.05 | 1.28 |
| Procr | 5.36 | 5.06 | 5.12 | 6.73 | 6.34 | 6.32 | + | 2.85 | 0.03 | 1.28 |
| Gm36962 | 0.87 | 0.92 | 0.95 | 1.96 | 2.43 | 2.40 | + | 3.05 | 0.02 | 1.35 |
| Rock2 | 8.58 | 8.39 | 8.41 | 9.48 | 9.76 | 10.24 | + | 2.40 | 0.05 | 1.37 |
| Hp | 8.68 | 9.04 | 8.44 | 9.90 | 10.14 | 10.30 | + | 2.57 | 0.04 | 1.39 |
| Arl4a | 3.19 | 2.92 | 3.70 | 4.72 | 4.75 | 4.58 | + | 2.42 | 0.05 | 1.41 |
| Dkk2 | 4.52 | 4.58 | 5.05 | 6.09 | 6.10 | 6.26 | + | 2.88 | 0.02 | 1.43 |
| Galnt17 | 4.00 | 3.92 | 4.08 | 5.13 | 5.27 | 5.91 | + | 2.38 | 0.05 | 1.44 |
| Klf13 | 5.26 | 5.57 | 5.37 | 6.81 | 6.84 | 6.88 | + | 4.05 | 0.01 | 1.44 |
| Nr1h3 | 2.68 | 2.99 | 2.53 | 4.13 | 4.17 | 4.26 | + | 3.30 | 0.01 | 1.45 |
| Arhgap6 | 3.26 | 3.81 | 3.85 | 5.36 | 4.90 | 5.07 | + | 2.50 | 0.04 | 1.47 |
| Lgalsl | 3.83 | 3.35 | 3.80 | 5.39 | 5.10 | 4.91 | + | 2.68 | 0.03 | 1.47 |
| Crispld2 | 5.82 | 6.29 | 5.91 | 7.59 | 7.70 | 7.24 | + | 2.76 | 0.03 | 1.50 |
| Map3k6 | 2.46 | 2.60 | 3.03 | 4.55 | 4.02 | 4.02 | + | 2.43 | 0.05 | 1.50 |
| Add3 | 5.61 | 5.51 | 5.65 | 7.05 | 7.07 | 7.17 | + | 4.97 | 0.02 | 1.51 |
| Kcnab1 | 2.33 | 2.60 | 2.41 | 4.13 | 3.88 | 3.89 | + | 3.73 | 0.01 | 1.52 |
| Gab3 | 3.33 | 3.81 | 3.41 | 5.05 | 4.94 | 5.33 | + | 2.96 | 0.02 | 1.59 |
| 2900005J15Rik | 1.20 | 0.92 | 0.95 | 2.55 | 2.70 | 2.74 | + | 4.00 | 0.02 | 1.64 |
| Prss23 | 5.18 | 4.77 | 5.32 | 6.52 | 6.82 | 7.03 | + | 2.81 | 0.02 | 1.70 |
| Slc1a5 | 5.94 | 5.55 | 6.34 | 7.95 | 7.55 | 7.53 | + | 2.55 | 0.03 | 1.73 |
| Postn | 7.83 | 7.98 | 8.29 | 10.16 | 9.85 | 9.33 | + | 2.50 | 0.03 | 1.75 |
| Hmgn5 | 5.94 | 5.46 | 5.14 | 7.09 | 7.43 | 7.30 | + | 2.65 | 0.02 | 1.76 |
| Ablim1 | 3.04 | 2.41 | 2.85 | 4.66 | 4.68 | 4.33 | + | 2.90 | 0.02 | 1.79 |
| Sparcl1 | 5.84 | 6.03 | 6.52 | 7.87 | 7.73 | 8.16 | + | 2.77 | 0.02 | 1.79 |
| Cp | 3.96 | 3.51 | 4.41 | 5.64 | 5.69 | 5.96 | + | 2.53 | 0.03 | 1.80 |
| Id4 | 5.68 | 5.42 | 5.49 | 7.10 | 7.39 | 7.60 | + | 3.44 | 0.02 | 1.84 |
| Mt1 | 8.15 | 8.29 | 7.93 | 10.34 | 9.75 | 9.85 | + | 3.04 | 0.01 | 1.85 |
| Col4a1 | 6.19 | 5.49 | 5.97 | 7.77 | 7.44 | 8.02 | + | 2.67 | 0.02 | 1.86 |
| Deptor | 5.10 | 5.46 | 6.04 | 7.26 | 7.44 | 7.51 | + | 2.55 | 0.03 | 1.87 |
| Dtna | 3.63 | 3.46 | 3.95 | 5.82 | 5.49 | 5.55 | + | 3.42 | 0.01 | 1.94 |
| Mitf | 4.00 | 3.35 | 3.95 | 5.80 | 5.59 | 5.86 | + | 3.06 | 0.01 | 1.98 |
| Stim1 | 5.30 | 5.67 | 6.28 | 7.95 | 7.70 | 7.57 | + | 2.54 | 0.03 | 1.99 |
| Khk | 2.46 | 2.30 | 2.53 | 4.42 | 3.99 | 4.87 | + | 2.79 | 0.02 | 2.00 |
| Cyth3 | 5.46 | 5.84 | 5.50 | 7.05 | 7.55 | 8.31 | + | 2.21 | 0.05 | 2.04 |
| Vit | 2.04 | 1.41 | 2.12 | 3.66 | 4.20 | 3.98 | + | 2.80 | 0.02 | 2.09 |
| Mt2 | 9.58 | 9.72 | 9.15 | 12.09 | 11.57 | 11.18 | + | 2.59 | 0.02 | 2.13 |
| Gramd1b | 4.00 | 4.41 | 4.78 | 6.19 | 6.31 | 7.11 | + | 2.38 | 0.03 | 2.14 |
| Gdap10 | 1.20 | 0.92 | 1.27 | 3.66 | 3.23 | 2.94 | + | 3.09 | 0.01 | 2.15 |
| Alpk3 | 2.46 | 1.18 | 1.53 | 3.77 | 3.81 | 4.26 | + | 2.25 | 0.04 | 2.22 |
| Fmo1 | 2.20 | 2.51 | 3.47 | 5.25 | 5.23 | 4.52 | + | 2.14 | 0.05 | 2.28 |
| Dusp14 | 0.45 | 0.60 | 0.54 | 3.55 | 2.61 | 2.64 | + | 2.82 | 0.01 | 2.40 |
| Adarb1 | 1.68 | 0.60 | 0.54 | 3.77 | 3.17 | 3.11 | + | 2.32 | 0.04 | 2.41 |
| Glul | 5.52 | 4.92 | 5.75 | 8.34 | 7.62 | 7.48 | + | 2.57 | 0.02 | 2.41 |
| Prrg3 | 2.96 | 2.19 | 3.27 | 5.64 | 5.25 | 4.82 | + | 2.44 | 0.03 | 2.43 |
| Fam46c | 0.87 | 0.92 | 0.54 | 3.66 | 2.99 | 3.26 | + | 3.40 | 0.01 | 2.53 |
| Nmb | 1.87 | 1.60 | 2.12 | 5.09 | 4.05 | 4.40 | + | 2.83 | 0.01 | 2.65 |
| Itgbl1 | 2.87 | 1.92 | 3.41 | 5.94 | 5.36 | 4.89 | + | 2.13 | 0.05 | 2.66 |
| Sult5a1 | 2.57 | 3.35 | 3.65 | 5.85 | 5.90 | 5.87 | + | 2.95 | 0.01 | 2.68 |
| Vldlr | 2.78 | 2.41 | 2.75 | 5.49 | 5.25 | 5.36 | + | 4.41 | 0.02 | 2.72 |
| Snta1 | 4.23 | 4.30 | 4.88 | 7.37 | 7.08 | 7.26 | + | 3.63 | 0.01 | 2.77 |
| Mchr1 | 1.87 | 2.06 | 2.27 | 4.36 | 5.05 | 5.24 | + | 3.19 | 0.01 | 2.82 |
| Dpf1 | 0.45 | 2.19 | 1.53 | 3.96 | 4.05 | 4.72 | + | 2.16 | 0.05 | 2.85 |
| Serpina3c | 1.46 | 0.60 | 1.27 | 4.66 | 4.05 | 3.46 | + | 2.61 | 0.02 | 2.95 |
| Scara5 | 4.92 | 5.38 | 5.77 | 8.08 | 8.64 | 8.21 | + | 3.24 | 0.01 | 2.96 |
| Rasgrp2 | 1.87 | 2.69 | 1.75 | 4.21 | 5.36 | 6.14 | + | 2.11 | 0.05 | 3.13 |
| Ace | 2.33 | 2.41 | 1.94 | 6.03 | 5.61 | 5.29 | + | 3.74 | 0.01 | 3.42 |
| Dpep1 | 8.18 | 8.53 | 8.93 | 11.43 | 11.99 | 12.58 | + | 3.02 | 0.01 | 3.45 |
| Eva1c | 0.45 | 1.92 | 0.54 | 4.36 | 4.31 | 4.64 | + | 2.68 | 0.01 | 3.46 |
| Wnk4 | 1.20 | 2.06 | 1.94 | 4.92 | 5.59 | 5.15 | + | 3.31 | 0.01 | 3.49 |
| Ndrg2 | 5.43 | 6.21 | 6.46 | 9.36 | 9.61 | 9.73 | + | 3.36 | 0.01 | 3.53 |
| Ramp2 | 5.50 | 5.65 | 5.52 | 8.74 | 9.30 | 9.27 | + | 4.34 | 0.00 | 3.55 |
| Hspb7 | 3.46 | 2.60 | 2.65 | 6.71 | 6.00 | 7.23 | + | 2.94 | 0.01 | 3.74 |
| Tsc22d3 | 3.63 | 3.18 | 3.95 | 8.02 | 7.43 | 6.71 | + | 3.01 | 0.01 | 3.80 |
| Ctla2a | 0.87 | 0.92 | 0.95 | 6.50 | 4.57 | 5.11 | + | 2.83 | 0.02 | 4.48 |
| Fmo2 | 1.46 | 0.19 | 1.75 | 5.72 | 5.24 | 6.05 | + | 2.98 | 0.01 | 4.53 |
| Map7d2 | 1.20 | 0.92 | 0.95 | 6.07 | 6.27 | 5.54 | + | 4.50 | 0.00 | 4.94 |
| Aldh1a1 | 1.46 | 1.18 | 2.95 | 7.56 | 7.14 | 7.27 | + | 3.20 | 0.01 | 5.46 |
